# Acoustic monitors and direct observations provide similar but distinct perspectives on bird assemblages in a lowland forest of eastern Ecuador

**DOI:** 10.7717/peerj.10565

**Published:** 2021-01-13

**Authors:** John G. Blake

**Affiliations:** Wildlife Ecology & Conservation, University of Florida, Gainesville, FL, USA

**Keywords:** Acoustic monitor, Avian, Community composition, Point count, Spatial variation, Temporal variation, Tropical, Vocalizations

## Abstract

Bird communities in lowland Neotropical forests exhibit temporal and spatial variation in species composition and abundance at multiple scales. Detecting and explaining such variation requires adequate methods for sampling those bird communities but counting birds in highly diverse lowland forests of the Neotropics can be particularly challenging. Point counts are one of the most frequently used methods for counting birds in tropical forests but inter- and intra-observer variability in detecting and identifying sounds may cause problems. Acoustic monitors (passive acoustic monitors; autonomous recording units) provide an alternative and potentially effective method to sample bird communities by acting, in effect, as “point counts”, recording vocalizations at a given point for a set time. I used acoustic monitors to examine patterns of species richness, spatial distribution, and community composition of birds in a lowland forest in eastern Ecuador, one of the most diverse regions on earth. I deployed monitors at 25 locations, each separated by at least 200 m, on each of two 100-ha plots (Harpia, Puma) at Tiputini Biodiversity Station during January–February, 2013–2017. Monitors were set to record for 10 min followed by a 5-min break, from 0545 h to 0810 h (10 recording periods/morning). Recordings were later reviewed to identify species; no attempt was made to distinguish individuals or to estimate distance. Results were compared with contemporaneous direct observations along transects on the same plots. A total of 214 species were identified from recordings on both plots, combined, with slightly more on Harpia (208) than on Puma (188). Number per year ranged from 142 on Harpia in 2016 to 161 on Puma in 2015. Number per point was ~45 with an overall range of 29–68. Number of species detected in recordings was similar to but somewhat less than the number recorded during direct observations. Number of species recorded increased rapidly from the first period (0545–0555 h) to the third (0615–0625 h) but showed little subsequent change. Most species were recorded at relatively few points; the four most widely distributed species were the same on both plots (*Patagioenas plumbea, Xiphorhynchus guttatus, Capito aurita, Ramphastos tucanus*), all of which are relatively loud canopy or subcanopy species. Ordinations based on species composition illustrated differences between plots based on both recordings and direct observations; similarly, patterns of species composition differed between methods. Acoustic monitors can be an effective tool for sampling bird communities and may be particularly effective and efficient for sampling loud species with distinctive songs. Nonetheless, results from monitors may provide different perspectives on species composition when compared to direct observations. Which method is preferred likely will depend on the specific objectives of individual studies.

## Introduction

Bird communities in lowland Neotropical forests exhibit temporal and spatial variation in species composition and abundance at multiple scales (Terborgh et al., 1990; Robinson, Brawn & Robinson, 2000; Blake, 2007; Menger et al., 2017). Temporal variation includes daily (hourly) changes in vocalization levels (Parker, 1991; Blake, 1992); seasonal variation as a consequence of movement patterns (Loiselle & Blake, 1991); and annual variation as species respond to changes in climate, habitat conditions, and other factors (Stouffer, 2007; Blake & Loiselle, 2015). Similarly, spatial variation may reflect small-scale differences in habitat structure and floristic composition (Robinson, Brawn & Robinson, 2000; Menger et al., 2017), changes in habitat across larger regional scales (Borges, 2004; Jankowski et al., 2009; Pomara et al., 2012), and changes across geographic scales (English, 1998; Robinson, Brawn & Robinson, 2000; Blake & Loiselle, 2009).

Detecting and explaining such differences in patterns of species composition and abundance is a major goal for ecology (MacArthur, 1972; Robinson, Brawn & Robinson, 2000; Pomara et al., 2012) but requires adequate methods for sampling bird communities (Terborgh et al., 1990). Although birds often are considered amenable to sampling (mostly diurnal, vocal, etc.), various factors can make sampling bird communities, especially those in highly diverse lowland forests of the Neotropics, difficult (Robinson, Lees & Blake, 2018). Point counts are one of the most frequently used methods for counting birds in tropical forests and elsewhere (Celis-Murillo, Deppe & Allen, 2009; Robinson, Lees & Blake, 2018) but suffer from various issues that can lead to erroneous results, including inter- and intra-observer variability in detecting and identifying sounds and in distance estimation (Robinson, Lees & Blake, 2018). Typically, only one point is sampled at a given time with a series of points sampled at different times of the morning; different species typically sing at different times (Parker, 1991; Blake, 1992; Hart et al., 2015) so time of count may influence which birds are detected at a given point.

Acoustic monitors (passive acoustic monitors, Deichmann et al. (2018); autonomous recording units, Shonfield & Bayne (2017)) have become increasingly used as an effective method to sample bird communities (see reviews by Blumstein et al. (2011) and Shonfield & Bayne (2017)). In effect, acoustic monitors may act as “point counts” by recording vocalizations at a given point for a set time, in the same way that observers do at a point (Darras et al., 2018). Comparisons between recorders and observers have shown that monitors may record more species than detected by an individual in some cases (Celis-Murillo, Deppe & Allen, 2009; Darras et al., 2018) but not in others (Leach et al., 2016; see reviews in Alquezar & Machado (2015), Shonfield & Bayne (2017), Darras et al. (2018) for more examples). Most comparisons are based on recordings that are made simultaneously with the observer’s count, with recordings reviewed later for species identification. Shonfield & Bayne (2017) provide a useful review of the uses of acoustic monitors in avian research as well as a discussion of the advantages and disadvantages of their use.

Here, I use acoustic monitors to examine patterns of species richness, spatial distribution, and community composition (based on species presence/absence) of birds in a lowland forest in eastern Ecuador. This site has been sampled for many years by direct observations (Blake, 2007; Blake & Loiselle, 2015) and a major goal of this study was to determine whether or not data from acoustic monitors would provide similar perspectives regarding the structure of the avian community. I examine how these acoustic parameters vary: temporally both within a morning (across different point counts) and across years; and spatially, among points within a plot and between study plots. I compare results based on monitors to those based on contemporaneous direct observations (sight and sound) along transects within the same study plots and across the same years.

## Methods

### Study site

Research was conducted at Tiputini Biodiversity Station (TBS), Orellana Province, Ecuador (*ca* 0°37′ S, 76°10′ W, 190–270 meters above sea level). TBS is located on the north bank of the Tiputini River, bordering Yasuní National Park and within Yasuní Biosphere Reserve, one of the most diverse regions of the world (Bass et al., 2010). The station and nearby areas are dominated by *terra firme* forest; *várzea* forest, palm swamps, and various successional habitats also are present. Mean annual precipitation at Yasuní Research Station, approximately 30 km WSW of TBS, is about 3,100 mm.

Two *ca* 100-ha plots (*ca* 1 km × 1 km each) were established in *terra firme* forest during 2001. Both plots are gridded (100-m east-west × 200-m north-south grid lines) and marked with 1.5-m PVC tubes at 50-m intervals. The Harpia plot ranges from ~201 to 233 m elevation and is characterized by more dissected upland forest. The Puma plot is flatter overall although elevation range is similar, from ~209 to 235 m. Flat areas on Puma may have pools of standing water after prolonged, heavy rains. Dominant vegetation on both plots is tall, evergreen forest although there are more areas of successional habitat (i.e., after tree blow-downs) on Puma.

### Bird sampling

**Figure 1:**
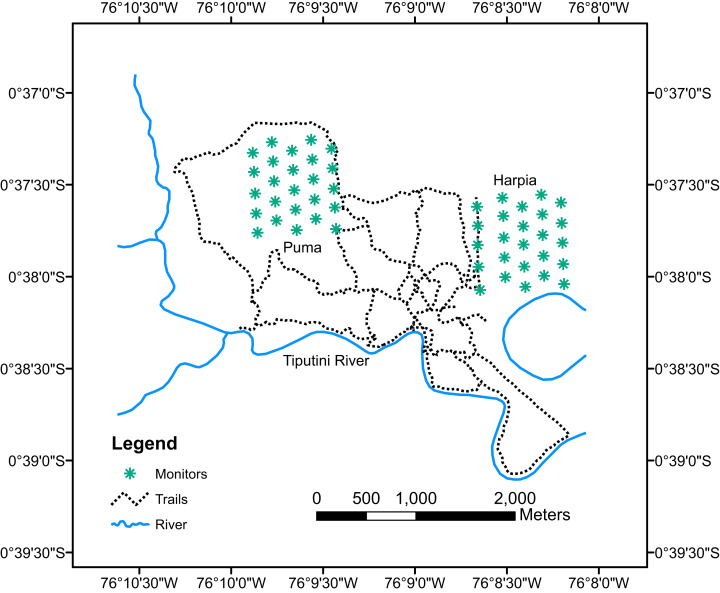
Map of Tiputini Biodiversity Station showing locations of monitors. Map of Tiputini Biodiversity Station showing locations of acoustic monitors on two 100-ha study plots, Harpia and Puma.

Birds were sampled during January–February, 2013–2017, with acoustic monitors (Song Meter SM2; Wildlife Acoustics, Inc., Maynard, MA, USA) equipped with two SMX-II omnidirectional microphones. Monitor failures and rain prevented complete sampling in some years, particularly on Puma plot. Monitors were attached to trees ~1.5 m above ground. Five monitors were deployed on each plot on transects located 200 m apart (e.g., on east-west transects 1, 3, 5, 7, and 9; Fig. 1). Monitors were left in place until two mornings without rain had elapsed and were then moved 200 m east (or west, depending on plot) to alternate transects 2, 4, 6, 8, and 10. Monitors were moved until 25 separate points were sampled on each plot (i.e., 10 mornings without rain). Monitors were set to record for 10 min followed by a 5-min break, starting at 0545 h and ending at 0810 h, for a total of 10 recording sessions (100 min) in a morning. Recordings were downloaded onto hard drives and subsequently manually reviewed to identify species; identifications were based on my knowledge of bird songs and calls and by comparisons to published songs and calls from birds in Ecuador. I also used Song Scope 4.1.5 (Wildlife Acoustics, Inc., Maynard, MA, USA) to visualize spectrograms of the different calls and songs, which aided identifications. No attempt was made to determine numbers of individuals recorded per species nor to estimate distance; thus, most analyses are based on numbers of species per recording period.

The most common auditory sensitivity of birds is ~1–6 kHz (Dooling, 2004). Suboscines are more likely to sing between ~1 and 6 kHz and oscines between 1 and 8 kHz with most suboscines between 1 and 5 and oscines 2–5 kHz (Weir, Wheatcroft & Price, 2012). Similarly, Aide et al. (2017) found that most bird vocalizations were less than 8 kHz. Given these considerations, I set monitors to record at a sampling rate of 16 kHz and 16 bits, providing a detection window up to 8 kHz, which encompassed the great majority of bird vocalizations, particularly those in the understory and louder canopy species. Although monitors likely missed some species, particularly canopy species with high frequency or quiet songs, they sampled most birds whose vocalizations were detectable and identifiable.

I also sampled birds with direct observations during February, 2013–2017 (i.e., during the same time periods that acoustic monitors were deployed). I recorded the locations of all birds seen or heard while walking along transects that covered each of the two sample plots; observations were not restricted to specific points (i.e., observations were not “point counts”). Approximately 0.9–1.2 km were covered in a morning; 10–12 days were required to sample each plot. Rain and other delays prevented complete sampling of plots in some years. Transects were not walked more than once during a given sample and starting locations were distributed throughout the plots to ensure that all parts of the plots were covered. Counts started well before light, when the first diurnal birds were beginning to sing and when many nocturnal species were still vocalizing. Vocal activity typically was high until ~2 h after sunrise, when it often declined rapidly; thus, counts were confined to the first few hours of the morning. Thus, acoustic monitors and direct observations sampled the same time periods. Further details of the observation procedures are in Blake (2007).

### Analyses

Numbers of species identified from recordings were summarized by point and time for one day of sampling per point per year. Time constraints precluded using both days of recordings. I used species accumulation curves and rarefaction to compare numbers of species recorded across all points within a plot for each year; rarefaction compared numbers of species recorded in different years based on the lowest number of total identifications between years being compared. Rarefaction was implemented with EcoSim Professional (Acquired Intelligence, Inc., 2012). When summarizing data from one time period (i.e., 10-min interval), I only counted a given species, including unidentified species, once no matter how many times the species vocalized during the count period. Number of individually identified records per point and number of species per point (summed across all counts, excluding unidentified vocalizations) were compared across years with repeated measures ANOVA, implemented with Statistix 10.0 (Analytical Software, 2013). Number of points at which an individual species was recorded was determined for each year; correlation analysis (Pearson’s *r*) was used to compare patterns of occurrence between years (i.e., to determine if number of points at which species were recorded was similar across years). I used the Bray–Curtis similarity index to compare composition of samples between sample time periods (e.g., between 0545 and 0600 h) within a given year; the index ranges from 1.0 (no difference) to 0 (completely different). I used non-metric multidimensional scaling (NMS) with the Bray–Curtis similarity index to compare composition of samples across years by sample method (acoustic monitors, direct observations) and location (Harpia, Puma). Species only recorded in one sample were omitted from the NMS analysis. Numbers of records from acoustic monitors and numbers of observations along transects were relativized prior to the analysis (general relativization by species and by samples; McCune & Grace, 2002). Relativization reduces the impact of very abundant species and focuses the analysis more on relative abundance patterns. Correlations between species and the first two axes of the ordinations were used to illustrate which species were most influential in distinguishing samples. Bray–Curtis similarities and NMS analyses were implemented with PC-ORD 6.0 (McCune & Mefford, 2011). NMS was followed by ANOSIM (analysis of similarities) which tests whether groups are more different from each other than expected by chance. ANOSIM was implemented with PRIMER 6, Version 6.1.6 (PRIMER-E, 2006).

### Approvals

Approval for this research was obtained from Institutional Animal Care and Use Committee, University of Florida Non-Regulatory Animal Research Committee (#201710065). Work at Tiputini Biodiversity Station was conducted in accordance with research permit number 025-2019-IC-PNY-DPAO (and earlier ones), Ministerio del Ambiente, Puerto Francisco de Orellana, Ecuador.

## Results

### Number of records from acoustic monitors

With records combined across count periods, I detected ~3,100 to ~3,600 separate records (i.e., number of species, both identified and unknown, detected summed across points; species were only counted once per time period no matter how many separate vocalizations were given by that species) per year during 5 years of sampling on Harpia (208 h of recordings) and ~2,800 to ~3,300 per year during 3 years on Puma (125 h; Table 1). Data from 2013 on Puma were not included in summary comparisons (e.g., number per year) because only 14 points were sampled; data from 2016 were not included as most recorders failed to work properly. Mean number of records per point ranged from a low of 114 on Puma in 2017 to a high of 143 on Harpia in 2014. Mean number per point did not differ among years on Harpia (*F*_4,96_ = 2.13, *P* = 0.083) but did on Puma (*F*_2,48_ = 4.84, *P* = 0.012, 2015 > 2017). Unidentified vocalizations accounted for ~5–6% of all records (Table 1).

**Table 1:** Number of records and species identified from acoustic monitors. Summary data on number of separate records (Rec’ds) obtained from acoustic monitors at 25 points on two 100-ha plots (Harpia, Puma) at Tiputini Biodiversity Station, Ecuador. Records unidentified to species (Unk) are included in totals (Rec’ds, Mean & SE/pt, Range) but not for number of species (Spp).

Plot	Year	Rec’ds	Mean/pt	SE/pt	Range	Unk	Spp	Sp/pt	SE/pt	Range
Harpia	2013	3,276	131.0	7.3	76–203	223 (6.8)	152	45.0	1.83	29–59
	2014	3,569	142.8	4.1	104–177	243 (6.8)	157	47.6	1.42	37–61
	2015	3,387	135.5	5.1	84–187	154 (4.5)	151	48.4	1.35	38–61
	2016	3,299	131.9	5.1	76–185	156 (4.7)	142	47.1	1.41	31–61
	2017	3,098	124.0	4.9	84–182	150 (4.8)	156	44.9	1.70	32–65
	Total species						208			
Puma	2013	2,098	149.9	9.2	100–210	178 (8.5)	135	51.0	2.57	35–68
	2014	3,183	127.3	6.3	62–186	160 (5.0)	143	45.4	1.69	29–61
	2015	3,335	133.4	6.5	73–191	188 (5.6)	161	46.9	1.84	32–63
	2016									
	2017	2,850	114.0	5.4	64–174	129 (5.0)	149	41.6	1.48	29–56
	Total species						188			

### Species richness

A total of 214 species were identified from recordings on both plots (Tables S1 and S2), combined, with slightly more on Harpia (208) than on Puma (188; Table 1). Number per year ranged from 142 on Harpia in 2016 to 161 on Puma in 2015. Number per point was ~45 with an overall range of 29–68 (Table 1). Number of species per point did not differ among years on Harpia (*F*_4,96_ = 1.6, *P* = 0.18) but did Puma (*F*_2,48_ = 4.85, *P* = 0.012, 2015 > 2017).

Number of species identified from recordings was similar to that detected while walking along transects (Table 2; Table S3). When compared on the basis of similar numbers of identified records (rarefaction analyses), direct observations (sight and sound) typically recorded more species (number identified > 95% CI for rarefied vocalizations from monitors; Table 2). Unidentified vocalizations accounted for many fewer observational records than based on monitors (Tables 1 and 2).

**Table 2:** Number of individuals and species observed on two study plots. Total number of individuals recorded during observation on two 100-ha plots (Harpia, Puma) and Tiputini Biodiversity Station. Number of days of observations are indicated for each year. Number of unidentified birds are given with percentage of total. Observed number of species is given as is the number expected based on rarefaction analysis (95% CI) of the monitor-based data using the same number of records.

Plot	Year (days)	Total	Unidentified (%)	Species	Expected number of species (95% CI)
Harpia	2013 (10)	1,447	14 (1.0)	149	[126–140]
	2014* (8)	996	6 (0.6)	151	[117–132]
	2015 (8)	1,159	2 (0.2)	152	[123–135]
	2016 (13)	1,331	11 (0.8)	147	[119–132]
	2017* (7)	747	30 (4.0	122	[109–125]
	Combined	5,680	63 (1.1)	212	
Puma	2013* (5)	595	8 (1.3)	143	[104–117]
	2014* (7)	879	13 (1.5)	143	[114–127]
	2015 (9)	1,112	25 (2.2)	153	[129–143]
	2016 (10)	961	34 (3.5)	146	
	2017* (7)	559	21 (3.8)	121	[103–117]
	Combined	4,106	101 (2.5)	206	

**Note:**

* Rains prevented complete sampling of plot.

**Figure 2:**
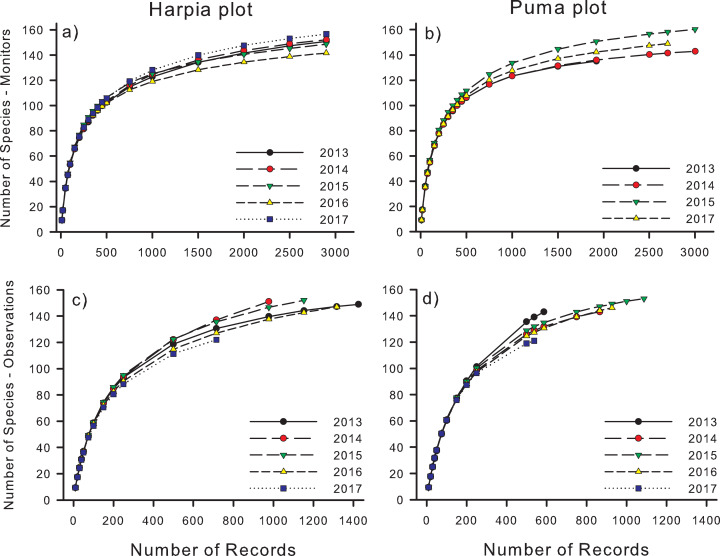
Species accumulation curves for recordings and direct observations. Species accumulation curves based based on species identified from acoustic monitor (A, B: Monitors) recordings and direct observations (C, D: Observations) on two 100-ha study plots (Harpia, Puma) at Tiputini Biodiversity Station, Ecuador.

Species accumulation curves increased rapidly to about 1,000 records but subsequently tended to level off, approaching but not reaching asymptotes (Fig. 2). Rarefaction analyses indicated that, on Harpia, species accumulation was greater during 2017 and less in 2016 than during the other 3 years, which did not differ from one another (Fig. 2A). In contrast, more species were accumulated during 2015 on Puma than during other years (Fig. 2B). When plots were compared by year, rarefied species totals were greater on Harpia during 2013, 2014, and 2017 and greater on Puma during 2015. Species richness based on rarefaction was higher based on direct observations in all years and on both plots except in 2017 on Harpia when curves did not differ (Figs. 2C and 2D; Table 2).

**Figure 3:**
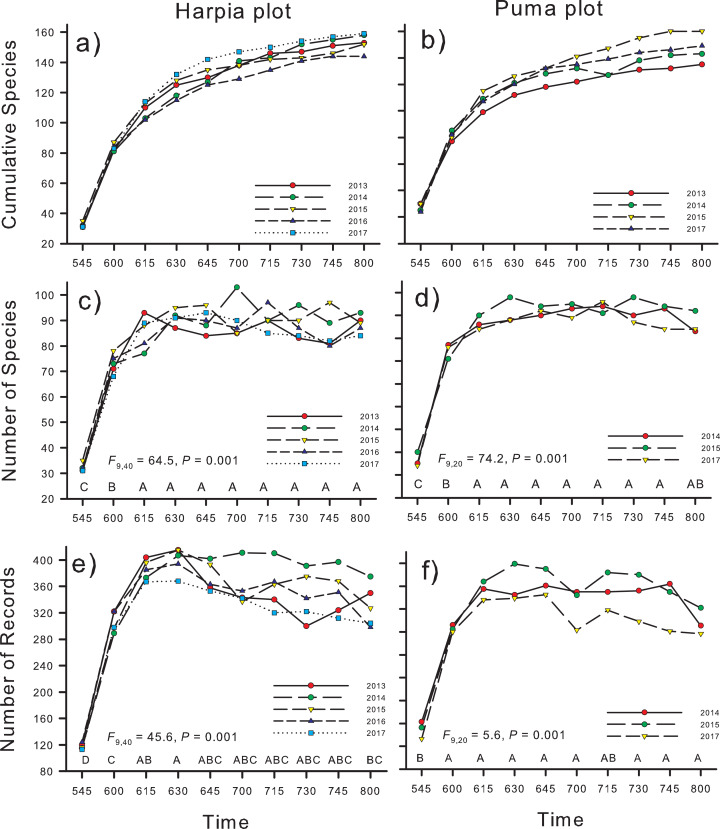
Numbers of species and records identified on two study plots, Tiputini Biodiversity Station, Ecuador. Numbers of species and numbers of records from acoustic monitors located on two 100-ha study plots, Harpia and Puma, at Tiputini Biodiversity Station, Ecuador. Cumulative number of species identified from 10 10-min recordings per point, starting at 0545 h and ending at 0810, combined across years (A and B); number of species identified during recordings made at different times in the morning, summed across 25 points (C and D); and number of records during the same count periods, summed across points (E and F). Results of ANOVA tests are given; time periods with the same letter did not differ in means.

Cumulative number of species increased rapidly from the first count (0545–0555 h; ~40 species total, points combined within a year) until 0630–0640 h when numbers reached ~120 species (Figs. 3A and 3B). In later counts, numbers continued to increase but more gradually. Number of species recorded during 10-min counts was significantly higher in the second count period than during the first and higher still during later counts (Figs. 3C and 3D; *F*_9,40_ = 64.5, *P* < 0.001, Harpia; *F*_9,20_ = 74.2, *P* < 0.001 Puma). Counts from 0615 to 0800 h did not differ in number of species recorded, on either plot. Number of vocalizations recorded followed a similar pattern except for a slight decrease during the last period (0800–0810 h) on Harpia (Figs. 3E and 3F; *F*_9,40_ = 45.6, *P* < 0.001, Harpia; *F*_9,20_ = 5.6, *P* < 0.001, Puma).

### Species distribution patterns

**Figure 4:**
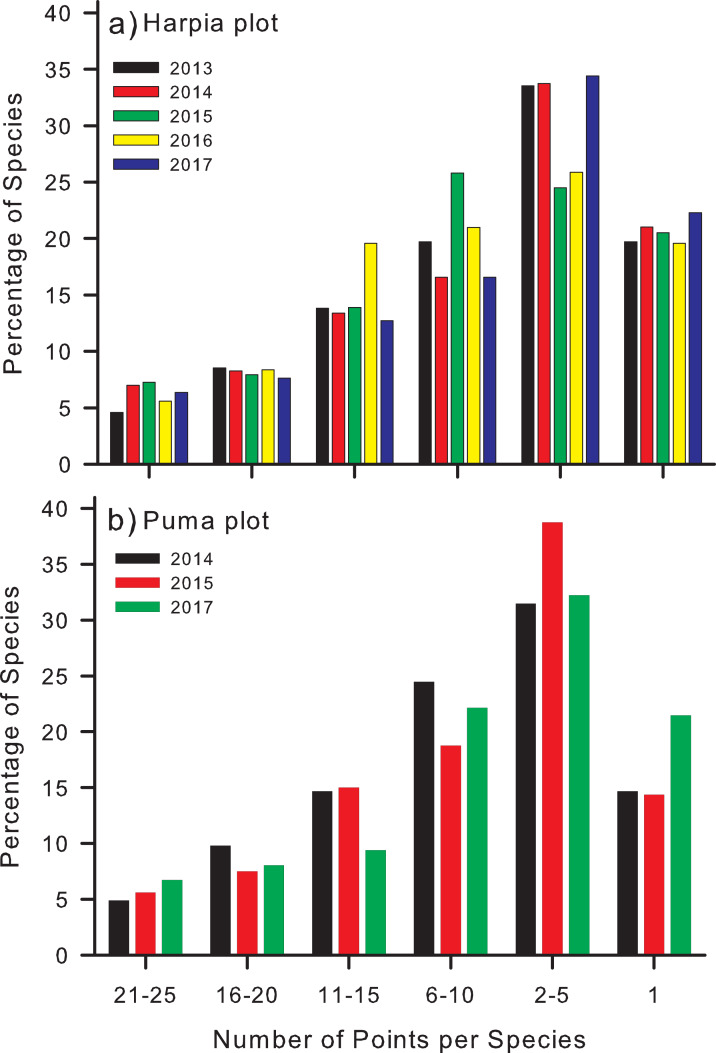
Percentage of points at which species were identified on two study plots. Percentages of species that were identified from recordings at different numbers of points (25 total) on each of two study plots, (A) Harpia and (B) Puma, Tiputini Biodiversity Station, Ecuador. Bars indicate results from individual years.

Most species were recorded at relatively few points during any 1 year (Fig. 4). Number recorded at 21–25 points (84–100% of points) varied from 7 to 11 on Harpia (Fig. 4A) and from 7 to 10 on Puma (Fig. 4B). In contrast, ~50% of all species were recorded on just 2 points (37–53 species on Harpia; 45–60 on Puma) or only 1 point (28–35 and 21–30, Harpia and Puma, respectively).

There were 38 species recorded at 15 (60%) or more points (all years combined) on Harpia and 32 on Puma (Tables 3 and 4; 5 years on Harpia, 3 on Puma). The four most widely distributed species (greatest number of points at which a species was recorded) were the same on both plots (*Patagioenas plumbea, Xiphorhynchus guttatus, Capito aurita, Ramphastos tucanus*), all of which are relatively loud canopy or subcanopy species. Fifteen species were among the 20 most widely distributed on each plot (Tables 3 and 4). Pairwise (between year) correlations based on number of points at which a species was recorded (species recorded at 15 or more points in at least 1 year) were significant for all comparisons on Harpia (*r* > 0.50, *P* < 0.001, all cases). Pairwise correlations on Puma were significant between 2014 and 2015 (*r* = 0.57, *P* < 0.001), between 2014 and 2017 (*r* = 0.74, *P* < 0.001), but not between 2015 and 2017 (*r* = 0.18, *P* = 0.30). Thus, overall, occurrence of species at points was generally similar across years.

**Table 3:** Species recorded at 15 or more points out of 25 on Harpia plot. Species recorded at 15 or more points (out of 25) on Harpia plot during at least 1 year (2013–2017). Number of points per year and mean across years is given.

Species	2013	2014	2015	2016	2017	Mean
*Patagioenas plumbea*	25	25	23	24	25	24.4
*Xiphorhynchus guttatus*	25	24	25	24	24	24.4
*Ramphastos tucanus*	25	23	22	25	22	23.4
*Capito auratus*	25	25	25	20	20	23.0
*Brotogeris cyanoptera*	20	24	22	24	24	22.8
*Amazona farinosa*	19	23	21	22	24	21.8
*Cercomacra cinerescens*	20	23	22	22	21	21.6
*Geotrygon montana*	18	24	23	19	22	21.2
*Thamnophilus murinus*	20	22	21	20	22	21.0
*Willisornis poecilinota*	19	19	23	19	21	20.2
*Glyphorynchus spirurus*	19	19	19	22	21	20.0
*Liosceles thoracicus*	21	20	16	20	17	18.8
*Thamnophilus schistaceus*	18	21	16	16	19	18.0
*Myrmotherula brachyura*	15	17	20	20	16	17.6
*Myrmoborus myotherinus*	23	19	17	12	16	17.4
*Trogon viridis*	14	18	23	18	13	17.2
*Ramphastos vitellinus*	12	15	20	23	16	17.2
*Hypocnemis cantator*	16	17	14	20	18	17.0
*Crypturellus variegatus*	18	19	17	11	14	15.8
*Cymbilaimus lineatus*	14	18	16	15	15	15.6
*Baryphthengus martii*	17	21	10	13	11	14.4
*Tolmomyias assimilis*	17	11	17	14	13	14.4
*Myrmothera campanisona*	16	14	15	10	17	14.4
*Campiphilus melanoleucos*	12	17	8	19	14	14.0
*Pionites melanocephalus*	9	14	10	17	19	13.8
*Saltator grossus*	13	15	10	13	17	13.6
*Otus watsoni*	13	13	11	14	17	13.6
*Psarocolius viridis*	23	3	7	20	13	13.2
*Atilla spadiceus*	12	12	15	12	14	13.0
*Lipaugus vociferans*	10	11	16	13	13	12.6
*Tinamus guttatus*	4	19	17	10	11	12.2
*Pygiptila stellaris*	12	12	15	12	10	12.2
*Tinamus major*	6	10	17	13	11	11.4
*Myrmeciza fortis*	15	13	10	10	7	11.0
*Trogon melanurus*	13	10	5	10	16	10.8
*Pipile cumanensis*	2	17	13	6	6	8.8
*Campiphilus rubricollis*	4	5	13	15	5	8.4
*Philydor erythropterum*	3	18	6	8	6	8.2

**Table 4:** Species recorded at 15 or more points out of 25 on Puma plot. Species recorded at 15 or more points (out of 25) on Puma plot during at least 1 year (2014, 2015 and 2017). Number of points per year and mean across years is given.

Species	2014	2015	2017	Mean
*Patagioenas plumbea*	25	25	24	24.7
*Xiphorhynchus guttatus*	25	24	25	24.7
*Capito auratus*	25	23	23	23.7
*Ramphastos tucanus*	25	21	22	22.7
*Campiphilus melanoleucos*	18	23	24	21.7
*Geotrygon montana*	24	23	14	20.3
*Baryphthengus martii*	23	19	19	20.3
*Ara macao*	16	22	22	20.0
*Myrmotherula brachyura*	19	21	19	19.7
*Brotogeris cyanoptera*	15	20	24	19.7
*Ramphastos vitellinus*	17	19	21	19.0
*Glyphorynchus spirurus*	21	18	16	18.3
*Psarocolius viridis*	20	21	12	17.7
*Wilisornis poecilinota*	19	18	16	17.7
*Tinamus major*	12	18	23	17.7
*Amazona farinosa*	12	17	23	17.3
*Cercomacra cinerescens*	18	15	17	16.7
*Myrmoborus myotherinus*	15	18	16	16.3
*Otus watsoni*	15	17	17	16.3
*Trogon viridis*	20	18	9	15.7
*Thamnomanes caesius*	16	15	16	15.7
*Tolmomyias assimilis*	14	17	16	15.7
*Thamnophilus murinus*	17	14	12	14.3
*Thamnomanes ardesiacus*	16	11	16	14.3
*Thamnophilus schistaceus*	13	16	13	14.0
*Crypturellus cinereus*	11	13	16	13.3
*Hypocnemis cantator*	17	10	12	13.0
*Mitu salvini*	19	11	8	12.7
*Myrmotherula axillaris*	15	14	9	12.7
*Amazona amazona*	7	11	16	11.3
*Pipile cumanensis*	18	9	6	11.0
*Sclerurus ruficollis*	15	6	2	7.7

### Community composition

**Figure 5:**
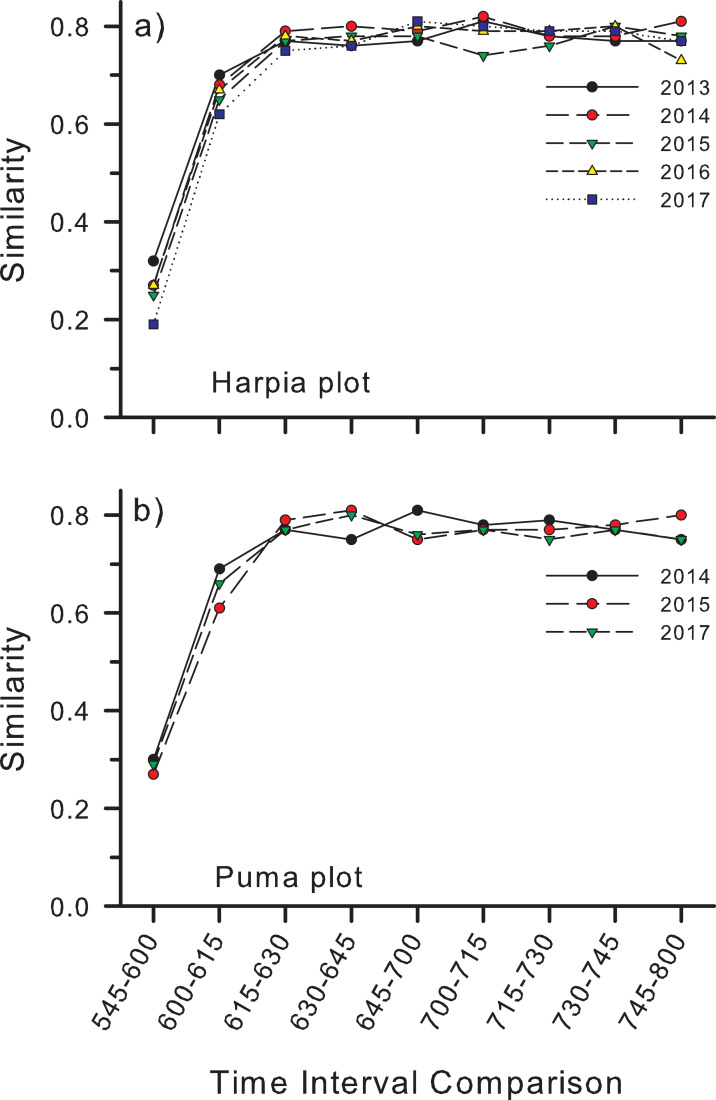
Similarity in species composition between different time periods in the morning. Similarity (Bray–Curtis index) in species composition between different time periods in the morning based on species identified from recordings made at 25 points on each of two study plots, (A) Harpia and (B) Puma, Tiputini Biodiversity Station, Ecuador.

Similarity (Bray–Curtis) in species composition between time intervals (e.g., between counts at 0545 h and 0600 h) followed a similar pattern to number of species per interval (Fig. 5). Similarity was lowest (~25–30%) between the first and second count periods on both plots, although slightly higher on Puma, higher between the second and third periods, and higher still and fairly constant among subsequent time intervals (Figs. 5A and 5B).

**Figure 6:**
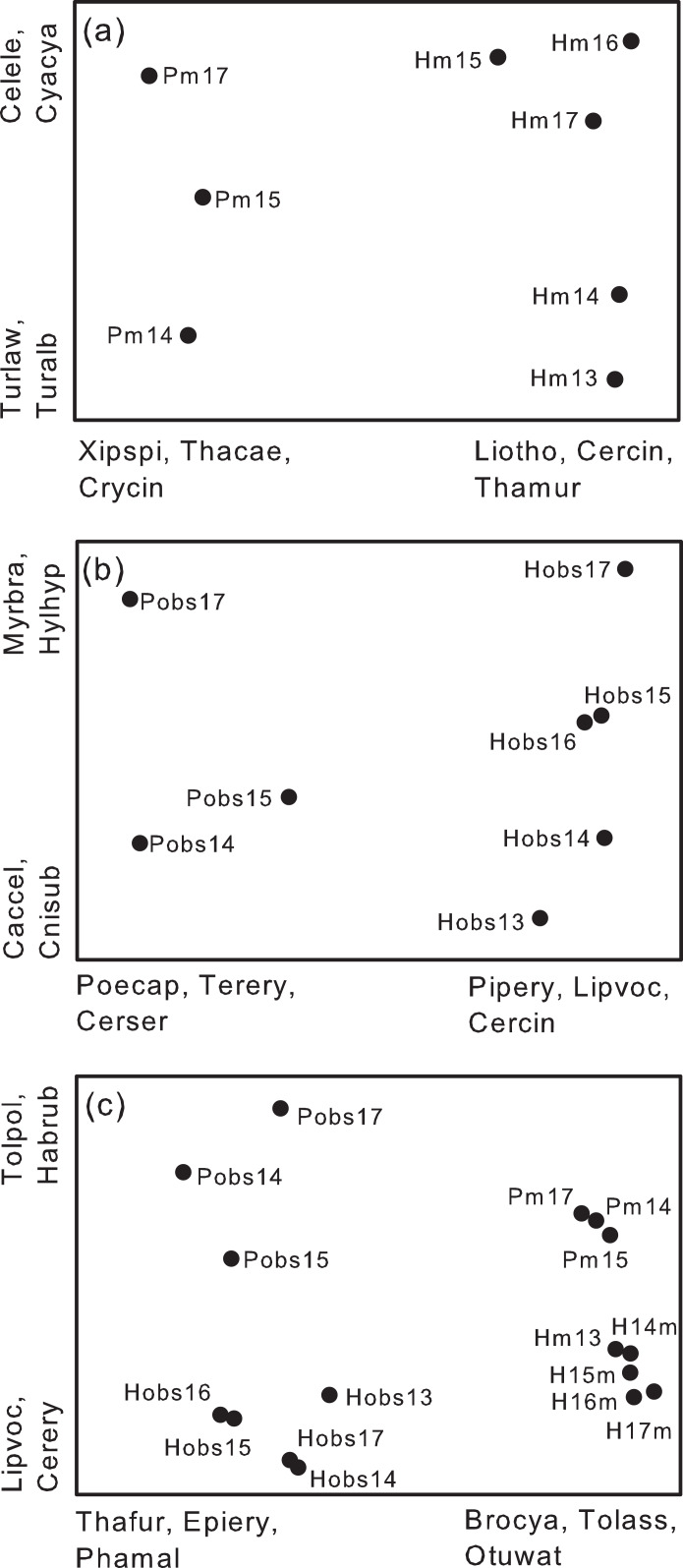
Non-metric multidimensional scaling ordinations based on species composition. Non-metric multidimensional scaling ordinations of yearly samples from two study plots (Harpia–H; Puma–P) at Tiputini Biodiversity Station, Ecuador. (A) Ordination based on recordings from acoustic monitors (m) at 25 points on each plot. (B) Ordination based on species identified during direct observations (obs) on each plot. (C) Ordination based on combined results from the two sampling methods. Species most highly correlated (negatively or positively) with the first two axes of each ordination are shown; codes reflect the first three letters of the genus and the first three of the species. Brocya, *Brotogeris cyanoptera*; Celele, *Celeus elegans*; Cercin, *Cercomacra cinerescens*, Cerser, *Cercomacra serva*; Cnisub, *Cnipodectes subbrunneus*; Crycin, *Crypturellus cinerescens*, Cyacya, *Cyanoloxia cyanoides*, Epiery, *Epinecrophylla erythrura*; Habrub, *Habia rubica*; Hylhyp, *Hylophilus hypoxantha*, Liotho, *Liosceles thoracicus*; Lipvoc, *Lipaugus vociferans*; Myrbra, *Myrmotherula brachyura*; Otuwat, *Otus watsoni*, Phamal, *Phaethornis malaris*; Pipery, *Pipra erythrocephala*; Poecap, *Poecilotriccus capitalis*; Terery, *Terenotriccus erythrurus*; Thafur, *Thalurania furcata*; Thacae, *Thamnomanes caesius*; Thamur, *Thamnophilus murinus*; Tolass, *Tolmomyias assimilis*; Tolpol, *Tolmomyias poliocephalus*; Turalb, *Turdus albicollis*; Turlaw, *Turdus lawrencii*; Xipspi, *Xiphorhynchus spixii*.

Overall species composition of yearly samples differed between study plots, despite the fact that many species were shared between the two plots (Fig. 6A). NMS ordinations omitted species recorded in only 1 year (18 species) leaving 196 species in the analysis. Species most highly correlated with the axes of the ordination differed between the two plots. For example, *Xiphorhynchus spixii, Thamnomanes caesius*, and *Crypturellus cinereous* were more frequently recorded on Puma whereas *Liosceles thoracicus, Cercomacra cinerescens, Thamnophilus murinus* and others were more frequently recorded on Harpia (Fig. 6A). These and other species clearly separated samples from the two plots along the first axis of the NMS. Species on the second axis were less highly correlated than along the first axis and tended to separate samples across years (e.g., *Celeus elegans* and *Cyanaloxia cyanoides* were more common in later years whereas *Turdus* spp. were more frequently recorded in earlier years; Fig. 6A). Results from ANOSIM indicated significant separation between plots (*R* = 0.98, *P* = 0.018).

Similarly, yearly samples from direct observations also clearly separated along the first axis of a separate NMS (Fig. 6B) based on an analysis with 193 species (44 omitted). ANOSIM confirmed a significant separation between plots based on direct observations (*R* = 0.95, *P* = 0.018). Species most highly correlated with the first axis overlapped somewhat with results from the acoustic monitors (e.g., *Poecilotriccus capitalis* and *Habia rubica* more associated with Puma, *Lipaugus vociferans* and *Cercomacra cinerescens* more associated with Harpia) but different species also were influential in separating the plots. For example, in contrast to *C. cinerescens*, the congener *C. serva* was more common on Puma (Fig. 6B). The second axis largely reflected differences in species composition among years. As with the first axis, there was some overlap with results from the monitors in terms of species most highly correlated with the second axis (Figs. 6A and 6B).

With data from observations and monitors combined (Fig. 6C; 226 species, 35 omitted), the first axis separated samples based on method whereas the second axis separated samples based on plot. Overall ANOSIM results indicated significant separation among groups (*R* = 0.97, *P* = 0.001). For example, observation-based samples had more hummingbirds (e.g., *Thalurania furcata*, *Phathornis boucieri*, *P. malaris*) whereas monitors recorded more *Brotogeris cyanocoptera*, *Tolmomyias assimilis*, and *Otus watsoni*, among others. *Lipaugus vociferans* and *Tyraneutes stolzmani* were more associated with Harpia whereas *Xiphorhynchus spixii*, *Habia rubica*, and *Poecilotriccus capitalis* were associated with Puma. As a consequence, there were four clearly defined clusters of samples in the ordination, based on both method and plot (Fig. 6C). When composition of plots was compared with methods combined, ANOSIM indicated a significant difference (*R* = 0.55, *P* = 0.001). Similarly, there was a significant separation between methods, with plots combined (*R* = 0.83, *P* = 0.001).

## Discussion

Acoustic monitors have proven to be useful for sampling bird species in a variety of habitats, including both temperate (Celis-Murillo, Deppe & Allen, 2009; Depraetere et al., 2011; Cook & Hartley, 2018) and tropical (Bueno et al., 2012; De Camargo, Roslin & Ovaskainen, 2019; Stevens et al., 2019) forests. They have been used to provide descriptions of bird species richness, community composition, and change in such communities over space and time (Towsey et al., 2014; De Camargo, Roslin & Ovaskainen, 2019; Stevens et al., 2019). Recordings from acoustic monitors also have been used to develop acoustic indices that provide information on diversity, without identification of individual species (Depraetere et al., 2011; Towsey et al., 2014; Aide et al., 2017). In the current study, acoustic monitors allowed me to investigate aspects of spatial and temporal variation in bird community composition within and between two 100-ha plots in lowland forest of eastern Ecuador. Spatial variation in species richness and composition was apparent at both within plot (i.e., among 25 sample points) and between plot scales (plots separated by about 1.5 km at the closest point). Temporal variation was apparent among point counts within a morning (i.e., from ~0545 to ~0810 h) and across years. Acoustic monitors and direct observations along transects provided similar estimates of species richness at the plot level, both on an annual basis and combined across years.

### Species richness

Species richness based on acoustic monitors was reasonably high with annual totals of around 150 species/plot. Numbers identified at an individual point also were high, ranging from about 30 to 70. This suggests that spatial overlap of species can be substantial with ~20–45% of plot total species richness occurring within sampling distance of a given monitor. Number of species identified on each plot from recordings was similar to that based on direct observations (based on rarefaction analyses), even though there were many fewer separate identifications from observations. Yet, the comparison is complicated by the fact that monitors recorded vocalizations at a given point for longer than time spent at a single location while walking transects. Thus, monitors certainly included multiple records of the same individuals (i.e., individuals that vocalized during more than one 10-min recording period).

Accumulation curves also revealed variation in species richness at both annual and hourly scales. Richness varied among years on both plots but not in a consistent manner; richness was greatest on Harpia in 2017 but on Puma in 2015 (comparison among years based on equal numbers of records), illustrating spatial variation in temporal patterns at the plot scale. Further, accumulation curves indicated that ~1,000 records were sufficient to provide an adequate description of species richness. Species richness also varied among counts during a morning, with the fewest recorded during the first count (~45 species) when it was still mostly dark. Numbers subsequently increased rapidly so that most species had been detected by about 0700 h. Similarity in species composition between count periods followed a similar pattern, with similarity lowest between the first few counts but fairly constant among later counts. This largely reflects the facts that (a) fewer species are vocalizing early in the morning and that (b) many of those early species do not continue to vocalize beyond the first few periods.

### Species composition

Acoustic monitors are effective at sampling many species, but they are not likely to provide a complete picture of species composition, particularly in species-rich lowland forests. Monitors worked well for species with clear, lower frequency vocalizations, particularly for those in the understory, but proved less effective for detecting canopy species, particularly those with relatively quiet and indistinct songs and calls, such as many tanagers, or that vocalize infrequently, such as many hummingbirds. Many of these species were detected during transect surveys that combined vocal and visual identifications. Monitors did, however, allow detection of some species with spotty distribution patterns or that vocalize infrequently that were missed during direct observations (e.g., *Notharchus macrorhynchus, Herpetotheres cachinnans, Sclateria naevia*), illustrating the value of combining methods. Leach et al. (2016) sampled birds in a rainforest in Queensland and found that point counts led to greater estimations of species richness because of additional species detected visually. Similarly, Stevens et al. (2019) found that point counts were more useful for canopy passerines in white-sand forests of Amazonia. On the other hand, Alquezar & Machado (2015) found that the two methods gave similar results in cerrado vegetation with visual detections at point counts not contributing additional species. Monitors and direct observations together may thus provide a more complete sample of species present in a given area, depending on the type of habitat.

Acoustic descriptions of species richness and composition depend on the number of detectable and identifiable vocalizations during sampling periods. In this study, numbers of detectable vocalizations were largely similar across years and between plots and typically exceeded 3,000 records; annual variation in numbers was significant on only one of the two plots (Puma). Of the vocalizations recorded, I could not identify approximately 5–6%, a larger share of the total when compared to direct observations (~1–2%). Species might be detected but not identified by a vocalization if it is too faint, not clear, or was a single call note. Such individuals might be identified during direct observations if the vocalization led to a visual detection.

Previous studies based on direct observations (Blake, 2007) and mist nets (Blake & Loiselle, 2009) demonstrated that the most common species were typically the same on both study plots (Harpia, Puma). Regional comparisons of results from 100-ha study plots in tropical forests also found many commonalities in the most dominant species (or congeners). In the current study, the same four species were the most widely distributed on each plot (*Patagioenas plumbea, Xiphorhynchus guttatus, Capito aurita, Ramphastos tucanus*), all of which produce relatively loud and easily identified vocalizations; 15 species were among the 20 most common on each plot. Further, between-year patterns in frequency of occurrence among points were generally similar, indicating that plots were characterized by a relatively consistent set of common species. Given the close proximity of the two plots (~1.5 km at closest point) and the fact that both are dominated by *terra firme* forest, such similarity is to be expected.

### Community composition

Despite the similarity in the identities of the most common species, the two plots nonetheless differed in overall species composition. NMS ordinations clearly indicated a separation between samples from the two plots, with annual samples from the same plot more similar to each other than to samples from the other plot, even after eliminating species recorded only once. Similarly, De Camargo, Roslin & Ovaskainen (2019) found that composition at a given point differed less between years than composition at different sites in the same year. The ordination also illustrated that composition changed across years in a similar way between plots—that is, direction of change in composition was similar even when the compositions differed between plots. Thus, it is important to consider both spatial and temporal variation in evaluations of community composition.

A similar overall pattern was seen when ordinations were based on results from direct observations—that is, plots separated along the first axis into two groups and separated along the second axis in response to changes in composition across years. When results from monitors and observations were combined, four distinct groups were found, reflecting differences between sampling methods as well as between plots and among years. That is, the species most important in separating annual samples between plots or years depended on the method used to sample plots. Hummingbirds, such as *Thalurania furcata* and *Phaethornis malaris*, with weak vocalizations, were not important for acoustic monitors but were important components of observations.

Many if not most species in tropical lowland forests are relatively rare and spatially restricted in distribution, often in response to small differences in habitat, topography, or other factors (Terborgh et al., 1990; Robinson, Brawn & Robinson, 2000; Blake & Loiselle, 2009; Bueno et al., 2012; Pomara et al., 2012; Menger et al., 2017). Results from acoustic monitors showed a similar result with about half of all species recorded from only 1 or 2 points on a given plot; many fewer were found at points throughout each plot. Similarly, at Cocha Cashu, Peru, 44 out of 245 species were found on at least 80% of the 100-ha plot but most species occupied much smaller areas (Terborgh et al., 1990).

Observations were conducted along transects that covered each plot in its entirety (any given spot is no more than ~50 m from a transect). Thus, some species detected along transects may have been associated with habitat conditions or locations not sampled by monitors. Many species can be detected at distances >100 m (e.g., *Lipaugus vociferans*) whereas others may not be detected at distances of ~50 m (e.g., *Platyrhynchus coronatus*) so monitors that were 200+ m apart could potentially miss individuals that were located between monitors. Monitors also may be more affected by interference from background noise (insects, primates) that make it difficult to identify vocalizations. Specific locations of monitors with respect to habitat or topography also may affect sound detection (Castro et al., 2019) and thereby influence estimates of species composition.

Acoustic monitors can be an effective tool for sampling bird communities and may be particularly effective and efficient for sampling loud species with distinctive songs (e.g., *Lipaugus vociferans*; Ulloa et al., 2016). Further, when studies focus on a select set of species with distinctive vocalizations, automatic detection software allows processing of many hours of recordings in a much shorter period than needed to manually listen to recordings (Acevedo et al., 2009; Aide et al., 2013; Ulloa et al., 2016; LeBien et al., 2020). Nonetheless, results from monitors also may provide a distinctly different perspective on overall community composition when compared to direct observations. Which method is preferred likely will depend on the specific objectives of individual studies. Monitors and direct observations differed in other aspects of sampling as well. By sampling points simultaneously, monitors allowed more detailed perspective on small-scale spatial variation in occurrence (i.e., among sample points) as well variation in temporal patterns of activity. Similarly, by simultaneously sampling replicate plots, monitors allowed examination of how richness and activity vary across slightly larger scales. Such simultaneous sampling at multiple points and plots typically is not possible with direct counts by observers.

## Supplemental Information

10.7717/peerj.10565/supp-1 Supplemental Information 1 List of species detected with acoustic monitors and/or by observations. Species detected on two study plots (Harpia - H, Puma -P) in lowland forest of eastern Ecuador at Tiputini Biodiversity Station, 2013-2017. Species detected by acoustic monitors at sampling points are indicated as H-pts or P-pts. Species detected by direct observations (sight or sound) are indicated as H-obs or P-obs. Taxonomy follows Remsen et al. 2020.

10.7717/peerj.10565/supp-2 Supplemental Information 2 List of species identified from recordings. Species identified from acoustic monitor recordings are listed by plot, year, point, date, and time. Species are identified by first three letters of genus and species names; see Table S1 for full names.

10.7717/peerj.10565/supp-3 Supplemental Information 3 List of species recorded along transects on two study plots. Species observed along transects on two study plots, Harpia and Puma, at Tiputini Biodiversity Station, Ecuador, 2013-2017 (raw data).

## Additional Information and Declarations

### Field Study Permissions

The following information was supplied relating to field study approvals (i.e., approving body and any reference numbers): Work at Tiputini Biodiversity Station was conducted in accordance with research permit number 025-2019-IC-PNY-DPAO (and earlier ones), Ministerio del Ambiente, Puerto Francisco de Orellana, Ecuador.

### Data Availability

The following information was supplied regarding data availability: Data are available in the Supplemental Files.
